# Eco-Friendly Combat against Prostate Cancer: Green Chemistry Approach Using Biosynthesized Nanoparticles Functionalized with Propolis for Enhanced Anticancer Activity

**DOI:** 10.32604/or.2025.070645

**Published:** 2026-03-23

**Authors:** Awatif Rashed Z. Almotairy, Eman Fayad, Fatimah Hadadi, Ahmad F. Alhomodi, Dalal Nasser Binjawhar, Hanadi A. Katouah, Bassma H. Elwakil, Keshav Raj Paudel, Mostafa El-Khatib

**Affiliations:** 1 Department of Chemistry, Faculty of Science, Taibah University, 30799, Yanbu, Saudi Arabia; 2 Health and Life Research Center, Taibah University, Madinah, Saudi Arabia; 3 Department of Biotechnology, College of Sciences, Taif University, P.O. Box 11099, Taif, 21944, Kingdom of Saudi Arabia; 4 Department of Biology, Faculty of Science, Al-Baha University, P.O. Box 1988, Al-Baha, 65527, Kingdom of Saudi Arabia; 5 Department of Biology, College of Science and Arts, Najran University, Najran, Kingdom of Saudi Arabia; 6 Department of Chemistry, College of Science, Princess Nourah bint Abdulrahman University, P.O. Box 84428, Riyadh, 11671, Kingdom of Saudi Arabia; 7 Chemistry Department, College of Science, Umm Al-Qura University, Makkah, 21955, Kingdom of Saudi Arabia; 8 Medical Laboratory Technology Program, Faculty of Applied Health Sciences Technology, Galala University, Suez, 43511, Egypt; 9 Centre for Inflammation, Centenary Institute, School of Life Sciences, Faculty of Science, University of Technology Sydney, Sydney, 2007, Australia; 10 Uttaranchal Institute of Pharmaceutical Sciences, Uttaranchal University, Dehradun, 248007, India; 11 Basic Sciences Department, Faculty of Computer Science and Artificial Intelligence, Pharos University in Alexandria, Beside Green Plaza Complex, Canal El Mahmoudia Street, Alexandria, 21648, Egypt

**Keywords:** Propolis, green chemistry, silver nanoparticles, multidrug resistance, anticancer

## Abstract

**Objectives:**

Prostate cancer cells often develop mechanisms to evade conventional therapies. Nanomedicine offers the potential for targeted drug delivery, improved tumor accumulation, and reduced systemic toxicity. This study biosynthesizes silver nanoparticles (NPP/AgONPs) functionalized with propolis, evaluates their antibacterial efficacy against uropathogenic strains of *Escherichia coli* (*E. coli*), and assesses their cytotoxic effect on cancer cell proliferation using the PC-3, human prostate epithelial cell line.

**Methods:**

The synthesized NPP/AgONPs physiochemical parameters were characterized, followed by *in vitro* assays to evaluate their antibacterial activity against multiple uropathogenic *E. coli* strains; determining the cytotoxicity against HPrEC and PC-3 cells by measuring cytotoxicity (CC_50_) and inhibition concentration (IC_50_), respectively; analyzing cell cycle distribution and apoptosis via flow cytometry; and quantifying the reactive oxygen species (ROS), Caspase 3, and Caspase 8 expression in treated cells to elucidate mechanisms of cell death and growth inhibition.

**Results:**

NPP/AgONPs exhibited an average particle size of 22 nm, with four major X-ray diffraction (XRD) peaks corresponding to Joint Committee on Powder Diffraction Standards (JCPDS) No. 01-1164, confirming their crystallinity. Moreover, the UV–vis absorbance at 390 nm yielded an energy gap of 2.45 eV. Antibacterial testing showed potent activity against the tested *E. coli* strains. In HPrEC and PC-3 cells, the CC_50_ was 262.04 µg/mL, while the IC_50_ was 25.34 μg/mL, respectively. Flow cytometry revealed increased apoptosis in the NPP/AgONPs-treated group across all stages, including early, late, and dead cells, compared with the controls. ROS, Caspase 3, and Caspase 8 levels were inflected in NPP/AgONPs-treated cells, showing apoptotic and growth-inhibitory effects.

**Conclusion:**

The propolis coating improves the nanoparticles’ biocompatibility while enabling potent ROS-mediated apoptosis and cell-cycle disruption in PC-3 cells. These findings support the potential of NPP/AgONPs as a synergistic therapeutic platform, though optimization of dosing, detailed mechanism elucidation, and assessment of long-term safety are warranted.

## 1 Introduction

The eco-friendly process for producing silver nanoparticles offers significant advantages over traditional chemical and physical methods. It uses reducing and stabilizing agents, such as fungi, plant extracts, and bacteria, which lead to lower toxicity, cost savings, and sustainability [1]. Unlike energy-intensive top-down methods (e.g., laser ablation) or chemical reduction techniques that use hazardous solvents, green methods produce fewer harmful by-products and lower energy consumption [2]. However, limitations include variability in nanoparticle size and shape due to the inconsistent nature of the biological precursors, slower production rates, and difficulties in scaling up for industrial use [3]. Conventional techniques, while providing better control over particle features and faster synthesis, pose greater environmental and health risks [4]. Thus, although green synthesis is a promising eco-friendly alternative, further improvement is needed to enhance consistency and scalability for commercial applications. The combination of green-synthesized NPP/AgONPs and propolis presents a new and sustainable antibacterial approach against prostate cancer.

The surface functionality of biologically produced NPP/AgONPs may affect their cytotoxicity against prostate cancer cells. Enzymes, proteins, amino acids, polysaccharides, vitamins, alkaloids, polyphenols, saponins, and other biomolecules play a significant role in the surface behavior of biogenic NPP/AgONPs, supporting the biological process of nanoparticle formation as stabilizing and reducing agents [5]. As these biomolecules continue to bind, they may reduce the cytotoxicity of the produced NPs [6]. There is a strong possibility to alter the function of NPs through surface functionalization, which involves attaching NPs to biological molecules like antibodies, aptamers, drugs, theranostic agents, and others. This opens up opportunities to develop multifunctional NPs with many potential applications, including theranostic systems, targeted drug delivery, and more [7].

NPP/AgONPs functionalized with propolis consistently demonstrate superior antibacterial activity compared to propolis alone, due to the synergistic effects of the silver core with propolis capping [8]. The underlying mechanism involves reactive oxygen species (ROS) modulation, disruption of microbial membranes, and sustained silver ion release enhanced by the propolis coating. However, chemically synthesized NPP/AgONPs formulations achieve similar activity, though biocompatibility and stability may vary. Likewise, the propolis coating potentially improves biocompatibility in non-tumor applications while maintaining anti-cancer effects, with stronger modulation of ROS and higher rates of apoptosis (early/late apoptosis) than propolis alone and chemically synthesized NPP/AgONPs formulations [9].

Despite a slow but steady decline in both incidence and fatality rates in recent years, prostate cancer remains one of the leading causes of death from malignant tumors worldwide [10]. Recent research increasingly supports the potential role of prostate microflora in the tumor microenvironment, particularly in the development, progression, and prognosis of prostate cancer [10]. Although its exact origins remain unknown, chronic prostatic inflammation is found in over 80% of benign prostates and is believed to significantly contribute to prostate carcinogenesis. Epidemiological studies have linked prostate cancer and prostatitis [11]. Uropathogenic strains of *Escherichia coli* and *enterococci* are often the main causal agents of chronic prostatitis, which can alter the prostate’s structure and even cause hyperplasia. These changes have been associated with decreased expression of the tumor suppressor NKX 3.1 in prostate tissue [12]. Additionally, evidence suggests that biofilm formation occurs in the prostate and other mucous membranes when bacteria from distant sites colonize the urinary tract via catheters [13]. Numerous microorganisms may infect the genitourinary tract through catheterization; however, research indicates that *Escherichia coli* is the most common [14]. Consequently, bacteria within biofilms are often highly resistant to antibiotics and the host’s immune defenses, facilitating bacterial invasion into host cells and tissues [15].

The present investigations aimed to produce silver nanoparticles from propolis extract to reduce potential toxicity and enhance their beneficial biological activity in treating prostate cancer and related microbes. The nano yield was analyzed to gather information about morphology, element composition, particle size, energy gap, and chemical composition using various tools, including high-resolution transmission electron microscopy (HR-TEM), scanning electron microscopy (SEM), Fourier transform infrared spectroscopy (FTIR), ultraviolet-visible (UV-Vis) spectroscopy, and Zeta potential.

## 2 Materials and Methods

### 2.1 Materials

Egyptian bee propolis was supplied by the Agricultural Research Center, Giza, Egypt. Tween-80 was procured from Thermo Fisher Scientific (Waltham, MA, USA). Streptozotocin (98% purity, CAS No. 18883-66-4) and citrate buffer solution (10 mmol/L, pH 4.5) were obtained from Sigma-Aldrich (St. Louis, MO, USA).

### 2.2 Propolis Extraction

Ten grams of propolis powder were dissolved in one hundred mL of absolute ethanol to make a propolis solution, which was then left to incubate for 6 h at room temperature [16,17]. The mixture was subsequently filtered through a 0.22 μm syringe filter (MilliporeSigma, Burlington, MA, USA) and stored at −4°C for further use [16,17].

### 2.3 Synthesis of Propolis Nanoemulsion

Propolis nanoemulsion was prepared using a low-energy thermal spontaneous emulsification method, as previously described with modifications [18]. Briefly, propolis powder was dispersed in double-deionized water containing Tween 80 (2% w/v) as a surfactant, followed by magnetic stirring (500 rpm, 1 h) and sonication (40 kHz, 15 min) (HH-S1, Zhengzhou Keda Machinery and Instrument Equipment Co., Ltd., Henan, China) [19]. The resultant emulsion was filtered through a 200 nm polyethersulfone (PES) membrane filter (6714-7502, Whatman, Cytiva, Marlborough, MA, USA).

### 2.4 Formulation of Silver-Loaded Propolis Nanoemulsion (NPP/AgONPs)

For the synthesis of NPP/AgONPs, 1 mL of 10% (w/v) propolis nanoemulsion was mixed with 9 mL of 5 mM silver nitrate (AgNO_3_, Sigma-Aldrich, St. Louis, MO, USA) under continuous magnetic stirring (800 rpm, 2 h) at 25 ± 2°C and pH 12 [20]. The formulated NPP/AgONPs were collected by centrifugation at 4500 rpm for 15 min, then decanted with an 80/20 ethanol–water mixture to remove any unreacted components, washed again with absolute ethanol, and finally dried at 25 ± 2°C. The formation of NPP/AgONPs was confirmed by visual observation of color change from yellow to brown and further characterized by UV-Vis spectroscopy as described in the characterization part.

### 2.5 Characterization

The produced nanosystem was separated from the solution using the centrifuge separation technique. Particles were separated based on their weight. A specialized cooling centrifuge (Hettich, MIKRO 2.0/2.0 R, Tuttlingen, Germany) is used. In this process, the heavier particles were forced to the bottom to form a dense lower layer. We then calculated the force exerted by the machine, known as the relative centrifugal force, using a standard formula that incorporates the centrifuge’s radius of 7.5 cm and its high rotational speed of 18,000 rpm. Chemical composition, size, shape, physical characteristics, crystallographic structure, and FTIR data were derived from:

#### 2.5.1 X-Ray Diffraction (XRD) Analysis

X-ray diffraction (XRD), where the instrument (Bruker D8 ADVANCE, Bruker, Karlsruhe, Germany) used a Cu-K_α_ (0.154 nm) monochromatic radiation to generate X-rays and was set to standard operating parameters of 30 kV and 30 mA. The analysis was performed at 25°C, with the detector scanning across a range from 0° to 100° in 2θ.

#### 2.5.2 HR-TEM Morphology and Size Analysis

A High-Resolution Transmission Electron Microscope (HR-TEM) (JEOL JEM-2100, JEOL Ltd., Tokyo, Japan) was used. A small droplet of the solution was placed onto a specialized copper grid that had a very thin carbon coating. We then simply let the droplet air-dry, leaving the nanoparticles firmly in place on the grid, ready for their structure and size to be examined. Image J free software license Version 1.54k produced by Bharti Airtel Ltd. (New Delhi, India) developer was used to automatically measure the spherical particles in HR-TEM image and calculate the average particle size of the prepared NPP/AgONPs.

#### 2.5.3 UV-Vis Spectroscopy and Plasmon Resonance

Ultraviolet (UV) was used to analyze the nanoparticles. We measured how they absorb light using a spectrophotometer (Thermo Scientific™ Evolution 300, Waltham, MA, USA), where 5 mL of each sample was placed into a special quartz cuvette and then analyzed using from 300 to 700 nm. This test was done to find the specific wavelength at which the particles absorb the light the most. The results successfully showed a distinct plasmonic peak around 350 nm, which is a recognized signature confirming the formation of NPP/AgONPs.

#### 2.5.4 Zeta Potential and Stability

Nano Zetasizer particle analyzer (nano ZS90; Malvern Panalytical Ltd., Malvern, UK), where the sample was dispersed in distilled water with a suitable viscosity of 0.8872 cP, and conductivity of 0.0899 mS/cm. 5 mL of the sample in a cuvette at five different pH levels from 2 to 8 were determined to get the point of zero charge pH.

#### 2.5.5 FTIR Functional Group Analysis

Fourier Transform Infrared spectroscopy (FTIR) analyses using a Shimadzu dxp 400 (Kyoto, Japan) gently mixed a small amount of nanoyield (about 2%) with potassium bromide powder and pressed the mixture into a thin, transparent pellet. This pellet was then immediately analyzed to capture its infrared absorption spectrum, measuring the unique molecular fingerprints across a key wavenumber range from 400 to 4000 cm^−1^ of the synthesized NPP/AgONPs.

#### 2.5.6 SEM-EDX Elemental and Microstructural Characterization

Energy dispersive X-ray (EDX) analysis was used as a comprehensive analytical platform; beyond basic elemental identification, it can capture digital images using Scanning Electron Microscope (SEM) (JEOL JSM-IT200, Akishima, Tokyo, Japan) and perform advanced crystallographic and orientation analysis through its integrated systems for electron backscattered diffraction and orientation imaging microscopy (Bruker EDX system, Berlin, Germany), operating at accelerating voltages up to 11 keV. Accurate information on elemental and mass ratio percentages can be easily obtained.

### 2.6 Antibacterial Assay

Using the conventional disc diffusion method, antibacterial activity experiments were performed against different strains of *Escherichia coli*. The various strains of *E. coli* were cultured in Luria-Bertani (LB) broth/agar medium (L2542, Sigma-Aldrich, St. Louis, MO, USA). The LB agar plates were inoculated overnight, and 100 μL of each culture was spread on the surface. A standard antibiotic disc was used for comparison with the tested nanoparticles, where a sterile paper disc with a diameter of 5 mm was loaded with 50 μg/mL of NPP/AgONPs in each plate [21]. To further investigate the antimicrobial properties of the prepared nanoparticles, researchers employed confocal scanning laser microscopy (CLSM) using a confocal scanner attached to a Leica DMI 6000 B FluoView microscope (TCS SP5) (Leica Microsystems, Wetzlar GmbH, Germany). The nanoparticles under test were introduced into the freshly prepared bacterial suspension. Confocal scanning laser microscopy was performed using a Kr/Ar laser (488 nm excitation) combined with a long-pass 514 nm emission filter. The green fluorescence signal and the red fluorescence signal were obtained by combining a 580 nm beam splitter with long-pass filters at 520 nm and 590 nm, respectively. Both green and red fluorescence were visualized using simultaneous dual-channel imaging with pseudocolor [22]. All data were presented as the average of three trials.

### 2.7 Cell Culture

The National Cancer Institute in Cairo, Egypt, provided the prostate cancer cell lines (PC-3, ATCC CRL-1435; Amelogenin: X, CSF1PO: 11, D13S317: 11, D16S539: 11, D5S818: 13, D7S820: 8,11, TH01: 6,7, TPOX: 8,9, vWA: 17, D3S1358: 16, D21S11: 29,31.2, D18S51: 14,15, Penta_E: 10,17, Penta_D: 9, D8S1179: 13, FGA: 24, D19S433: 14, D2S1338: 18,20; with no mycoplasma contamination) and normal Primary Prostate Epithelial Cells; Normal, Human (HPrEC) (ATCC PCS-440-010). Following a methodology established by the National Cancer Institute, the cells were cultured in RPMI-1640/DMEM with L-glutamine supplied by Lonza Verviers SPRL of Verviers, Belgium. Fetal bovine serum (FBS; Sigma-Aldrich, St. Louis, MO, USA) and penicillin-streptomycin (10% and 1%, respectively) (Sigma-Aldrich, St. Louis, MO, USA) were added to the cell culture medium. All of the cell batches were incubated at 37°C with 5% CO_2_. A density of 5 × 10^3^ cells per well was used when the cells were seeded onto 96-well plates three times [23].

#### 2.7.1 Cell Viability Assay

We examined how different concentrations of the formulated NPP/AgONPs impact the PC-3 and HPrEC normal prostate epithelial cells’ viability using the MTT colorimetric assay. Both cells were seeded on a well plate and cultured overnight at 37°C with 5% CO_2_, followed by treatment with different doses of NPP/AgONPs for 24 h. The formazan crystals that had developed after incubating the cells with MTT reagent for 3 h were dissolved in DMSO. At 570 nm, the absorbance was measured using a microplate reader. The fraction of dead cells is inversely linked to the purple formazan color intensity.

#### 2.7.2 Intracellular ROS Measurement

Due to reactive oxygen species’ (ROS) central role in both cancer cell development and death, intracellular ROS levels were measured in NPP/AgONPs-treated PC-3 cells. The fluorescent dye, 2,7-diacetyl dichlorofluorescein (DCFH-DA staining) (287810, Sigma-Aldrich, St. Louis, MO, USA), was used in measuring the intracellular ROS levels. Various concentrations of NPP/AgONPs were added to the PC-3 cells and incubated at 37°C for one day. After adding 1 μL of DCFH-DA (1 mg/mL), the fluorescence intensity was measured at excitation wavelength 485 and emission wavelength 530 nm using a fluorescence microplate reader (Synergy H1, Agilent Technologies, Winooski, VT, USA).

#### 2.7.3 Caspase 8, 9, and 3 Activity Assay

Apoptosis is carried out by proteolytic enzymes called caspases. To explore if NPP/AgONPs induce apoptosis of PC-3 cells, we measured the levels of caspases using an assay kit from Invitrogen (V35117, Thermo Fisher Scientific, Waltham, MA, USA) to measure the activity of caspases 8, 9, and 3. After being exposed to varying concentrations of NPP/AgONPs, PC-3 cells were incubated at 37°C for 24 h. Next, the caspase estimate was performed on both the control (NPP/AgONPs untreated) and treated cells according to the manufacturer’s instructions at 405 nm [24].

#### 2.7.4 Cell Cycle Analysis Assay

An apoptosis detection kit called Annexin V-FITC/Propidium Iodide (PI) was used to evaluate cell death (ab139418, Abcam, Cambridge, UK). The PC-3 cells that were collected (1 × 10^6^) were immersed in 66% ethanol for 2 h before being kept at 4°C. After that, the cells were washed with 1× Phosphate Buffered Saline (PBS) at pH 7.4 and left to stain with RNase and propidium iodide for 1/2 h. The results were examined using a flow cytometer (BD FACSCalibur, BD Biosciences, San Jose, CA, USA) after measuring the propidium iodide fluorescence intensity on FL2 with a 488 nm laser excitation (Excitation maximum, 493 nm; Emission maximum, 636 nm) [25]. Briefly, flow cytometry data were analyzed using FlowJo (Biosciences, San Jose, CA, USA). The gating strategy was applied sequentially to all events collected to ensure accurate analysis of viable, single cells for DNA content. Firstly, Debris Exclusion (FSC/SSC Gating) step: Initial gating was performed using a forward scatter (FSC-A) vs. side scatter (SSC-A) density plot. A broad polygonal gate was applied to the main population of cells. Secondly, Viability Gating step: To further ensure the analysis focused on healthy cells, the events within the R1 gate were assessed for viability using the incorporated viability dye signal (Propidium Iodide). Dead cells, which typically exhibit altered morphology and higher/lower viability dye uptake depending on the dye type, were excluded from subsequent analysis. Followed by the Doublet Discrimination (Single Cell Gating) step: To ensure that each event represented a single cell and to prevent misinterpretation of cell aggregates as G2/M phase cells, doublet discrimination was performed. The viable population was plotted using FSC-Area (FSC-A) vs. FSC-Width (FSC-W) parameters. A narrow, tight gate was drawn around the linear diagonal population, which represents single cells. Events outside this gate, identified as doublets or aggregates, were excluded from the final DNA content analysis. Finally, Cell Cycle Analysis: The final, gated population of live single cells was used for cell cycle analysis. A histogram of DNA content (fluorescence intensity of propidium iodide) was generated and analyzed using FlowJo’s cell cycle module (Biosciences, San Jose, CA, USA) to quantify the percentage of cells in the G0/G1, S, and G2/M phases of the cell cycle.

### 2.8 Statistical Analysis

All results are reported as the means of at least three independent replicates. Data handling and analysis were performed using SPSS Statistics version 24 (IBM Corp., Armonk, NY, USA). Outcomes are expressed as mean ± standard deviation (SD). Intergroup comparisons employed a one-way ANOVA to detect differences among groups, followed by Tukey’s post hoc tests for pairwise contrasts. A *p*-value < 0.05 was deemed statistically significant.

## 3 Results

### 3.1 Characterization

**Figure 1:**
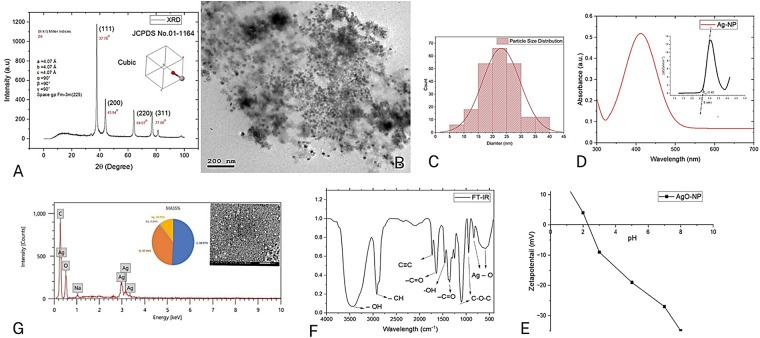
Characterization and properties of NPP/AgONPs. (**A**) XRD spectra. (**B**) HR-TEM (200 nm). (**C)** size distribution. (**D**) UV analysis and Tauc plots. (**E**) pH of the zero point of charge (pH_ZPC_). (**F**) FT-IR analysis. (**G**) EDX analysis (500 nm)

The nano yield in powder form was produced and separated from the aqueous solution using a centrifuge method. *XRD* data phase Analysis by X-ray diffractometer using Cu_Ka_ radiation. performed as shown in Fig. 1A at the nano yield to examine the average crystallite size $D_{XRD}$ (≈10.9 nm) for the four major peaks mentioned in Fig. 1A by the Debye-Scherrer equation [26,27] as mentioned in Eq. (1):

(1) $$D_{XRD}=\frac{\alpha \lambda }{\beta cos\theta },$$

where *β* seems to be the full width of half maximum (FWHM), *λ* denotes X-ray wavelength (1.5406 Å), *α* Scherrer constant (0.9), and 2*θ* for Bragg’s angle. To calculate the micro-strain (*ε*), Eq. (2) was used [27]:

(2) $$\varepsilon =\frac{\beta cos\theta }{4}$$

In conformity with HR-TEM findings, the developed silver nanoparticles have a face-centered cubic (FCC) structure with a space group of Fm-3m (255), as shown by the high intensity peak (111).

The following equations have been utilized to evaluate the specific surface area calculated ($SSA_{Calc.}$) and the dislocation density (δ) are given by using crystallite diameter calculated Eqs. (3) and (4), respectively [27]:

(3) $$\begin{array}{rl} & \delta =\frac{1}{{D_{XRD}}^{2}} \end{array}$$

(4) $$\begin{array}{rl} & SSA_{Calc.}=\frac{6000}{\rho_{x.}D_{XRD}} \end{array}$$

in which *ρ*_*x*_ seems to be the X-ray density. A crystal’s dislocation density is determined by the length of its dislocation lines per unit of volume, which is usually affected by the size of vacancies and defects existing in the crystal [27].

Crystallinity index $I_{cry}$ is measured by comparing the particle sizes found by *HR*-*TEM* $\left(D_{HR-TEM}\right)$ and crystallite size measured by *XRD* $\left(D_{XRD}\right)$. To get the crystallinity index, one uses Eq. (5):

(5) $$I_{cry}=\frac{D_{HR-TEM}}{D_{XRD}}$$

where A_A_ and A_C_ stand for the respective measured data of the region under all diffraction peaks and the crystal peak.

HR-TEM images obtained in Fig. 1B showed a uniform spherical shape of the synthesized NPP/AgONPs with a diameter of around 22 nm. Fig. 1C, as calculated by the Image J software size distribution program, agrees well with XRD results.

In this section, UV-Vis spectroscopy was employed to analyze the morphology, particle size, and yield of the synthesized nanoparticles [26]. The absorbance UV-Vis spectra of the prepared yield showed a single plasmonic peak at a wavelength of 390.23 ± 0.76 nm, as shown in Fig. 1D. The sensitivity of UV-Vis spectroscopy is strongly influenced by the nanoparticle shape. As illustrated in Fig. 1D, the observed absorbance peaks were broad and singular, suggesting the formation of spherical silver nanoparticles [26,27].

The FTIR spectrum in Fig. 1F (400–4000 cm^−1^) highlights the vibrational peaks of the propolis-synthesized NPP/AgONPs. Characteristic bonds, such as C-O, C-H, and C-C, are visible, consistent with prior findings [11]. The broad absorption between 3425–3450 cm^−1^ corresponds to O-H stretching from the aqueous sample preparation, supporting the presence of phenolic compounds in the propolis extract. Key functional groups involved in binding interactions were identified, with shifts in intensity or wavenumber providing further insight. The 2932 cm^−1^ peak signifies C-H stretching, suggesting the existence of long-chain alkyl compounds in the propolis extract. The 1614 cm^−1^ band indicates N-H bending, likely from glycosides in propolis. Aromatic terpenoid saponins are evident at 1522 cm^−1^, while the 1445 cm^−1^ peak confirms carboxylate groups in the propolis extract. The 1381 cm^−1^ band reflects C-H bending in glucose-derived aldehydes, and the 1258 cm^−1^ peak suggests ester linkage (C-C(=O)-O) stretching. C-O stretching is clearly visible at 1072 cm^−1^, and the 530 cm^−1^ band confirmed the crystal structure of NPP/AgONPs associated with hydroxyl group interactions from the propolis extract. The peak at 830 cm^−1^ might be due to the bonding of the C-H group with NPP/AgONPs.

The zeta potential analysis of the prepared sample, conducted with a Zetasizer Nano ZS 90, showed a negative surface charge Fig. 1E, which is attributed to the particle’s electrostatic properties at normal pH.

To determine the elemental composition of our sample, we performed EDX analysis Fig. 1G. The results showed a high concentration of carbon and oxygen—the primary components of the organic propolis extract—along with a significant amount of silver. The detection of silver, alongside trace amounts of other elements like sodium (under 1%), strongly supports the successful formation of silver nanoparticles (NPP/AgONPs) attached to the propolis extract.

### 3.2 Antibacterial Effect

**Figure 2:**
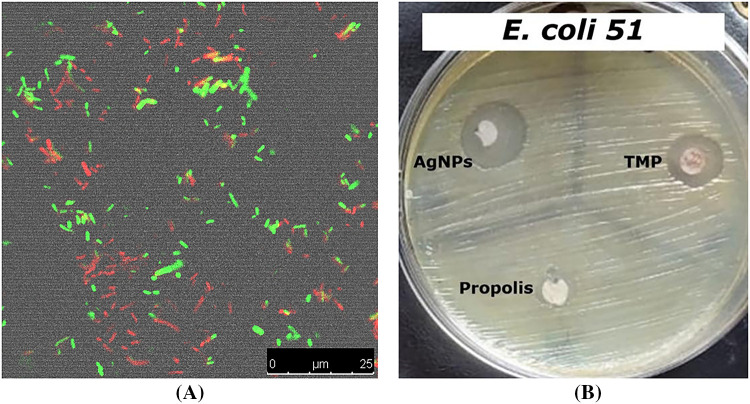
Antibacterial activity assessment of the synthesized NPP/AgONPs using CLSM (**A**) and disc diffusion (**B**) studies against *E. coli* 51

Results revealed that NPP/AgONPs have a strong antibacterial effect against several *E. coli* strains compared to the standard testing antibiotic. *E. coli* 51 was the most resistant strain; hence, it was chosen for further antibacterial studies (Table 1 and Fig. 2B). In order to determine the proportion of growth decrease associated with an increase in red (dead) cells compared to green (viable) cells, the live/dead cell experiment was evaluated using confocal scanning laser microscopy (CLSM) (Fig. 2A).

**Table 1:** NPP/AgONPs’ antibacterial activity against the tested *E. coli* strains

Tested bacteria	Inhibition zone diameter (mm)	
	Trimethoprim	NPP/AgONPs
*E. coli* 15	13.0 ± 0.0	20.0 ± 1.5
*E. coli* 17	10.0 ± 1.0	19.0 ± 0.5
*E. coli* 29	12.0 ± 1.0	20.0 ± 1.0
*E. coli* 36	11.0 ± 2.0	19.0 ± 0.0
*E. coli* 40	10.0 ± 0.5	18.0 ± 0.5
*E. coli* 51	10.0 ± 1.0	17.0 ± 0.5
*E. coli* 59	15.0 ± 0.0	19.0 ± 2.0

### 3.3 Anti-Prostate Cancer Effect

**Figure 3:**
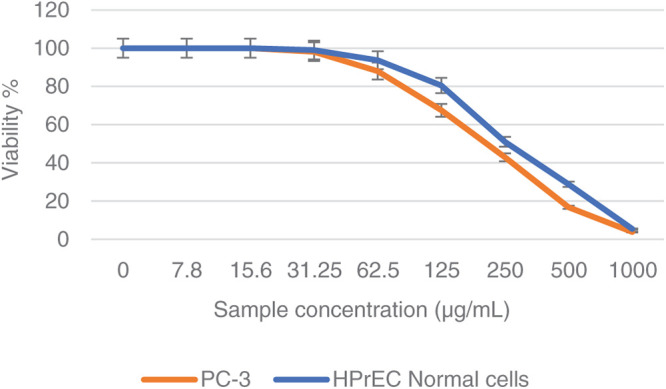
Cell viability % against NPP/AgONPs-treated cells

**Figure 4:**
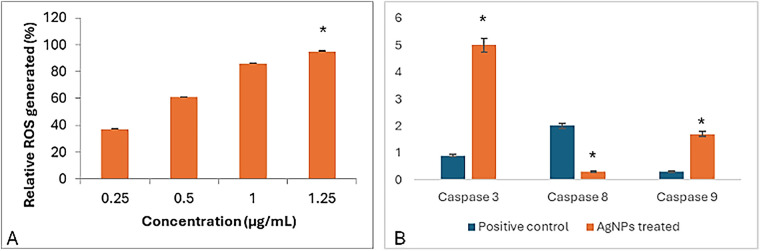
Apoptotic effect of the synthesized NPP/AgONPs, where (**A**) ROS and (**B**) cas-3, 8, and 9 biomarkers expression. *p < 0.05, analyzed using Student’s *t*-test. In Fig. 4A, a significant difference was found between the lowest and the highest concentrations

**Figure 5:**
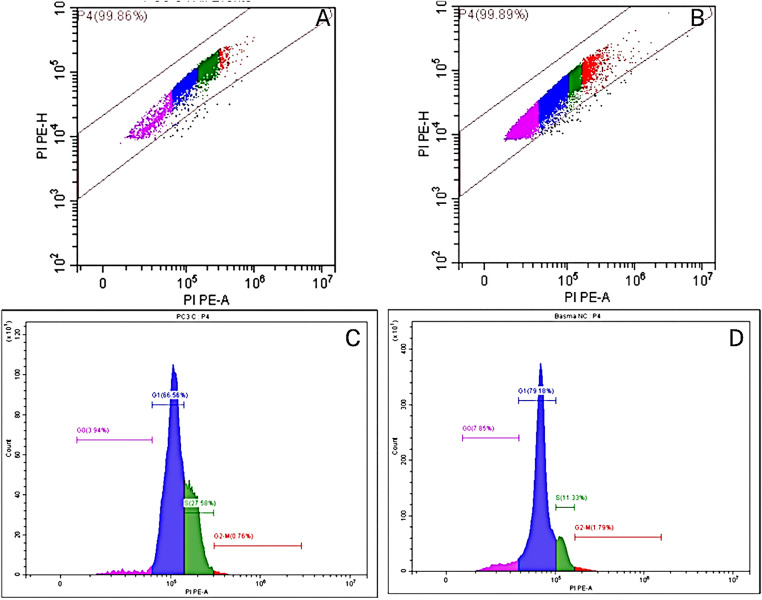
Flow cytometry plots in control (**A**) and treated (**B**) PC-3 cells using the Annexin V-FITC/PI double labeling approach, and flow cytometric profile of control (**C**) and treated (**D**) PC-3 cells

In a trial studying the *in vitro* cytotoxic effect of the biosynthesized NPP/AgONPs, cell proliferation using the human prostate epithelial cell line PC-3 was investigated. It was found that with 15.6 μg/mL of NPP/AgONPs, the PC-3 cell viability was 100.0%, while the CC_50_ of HPrEC normal prostate epithelial cells reached 262.04 μg/mL (Fig. 3). On the other hand, the IC_50_ against PC-3 cells reached 25.34 μg/mL (Fig. 3). Since reactive oxygen species (ROS) are known to be involved in every cellular process (cell survival, proliferation, differentiation, protein synthesis, and inflammation) and play a pivotal role in cancer development, we examined the expression of ROS, Caspase 3, and Caspase 8 (Fig. 4). The produced nanoparticles showed strong anti-cancer properties in PC-3 cells. Also, flow cytometry was utilized to examine how often the cell cycle occurred in PC-3 cells as a result of NPP/AgONPs. Table 2 and Fig. 5A–D show that the experimental group showed PC-3 cell apoptosis, with a significant increase in early and late apoptosis as well as necrosis, leading to death. Interrupting cell division is associated with suppressing cell growth.

**Table 2:** NPP/AgONPs-cell cycle progression in PC-3 cells

Cells	%G0–G1	%S
Control PC-3	66.47	27.54
NPP/AgONPs-treated cells	79.09	11.32

## 4 Discussion

Propolis-functionalized silver nanoparticles combine the natural bioactivity of propolis with the antimicrobial and physicochemical advantages of silver nanomaterials. They are typically stable, spherical, and highly effective against a range of pathogens, with additional antioxidants and wound-healing properties. These features support their potential in medical, pharmaceutical, and environmental applications, though further safety and cytotoxicity studies are needed for clinical translation. In consistent with the characteristics of the currently prepared nanoparticle, a previous study detailed that it was determined that the obtained silver nanoparticles had a maximum UV absorbance at 425 nm and their sizes ranged from 67 to 75 nm [28]. Barbosa et al. [29] stated that AgNP-P (silver nanoparticles synthesized with propolis extract) demonstrated a maximum absorbance at 412 nm in ultraviolet-visible spectra. Dynamic light scattering demonstrated a hydrodynamic size of 109 nm and a polydispersity index of less than 0.3, showing a good size distribution and stability. After its purification, microscopy analysis corroborates the format and shows the presence of propolis around the silver nanoparticle. X-ray diffraction peaks were attributed to the main planes of the metallic silver crystalline structure; meanwhile, infrared spectroscopy demonstrated the main groups responsible for silver reduction, represented by ∼22% of AgNP-P indicated by thermal analysis. Another study revealed that the sizes of the synthesized silver nanoparticles were 9.43 and 11.39 nm, according to TEM and XRD analyses, respectively. A negative Zeta potential of −8.8 mV was obtained, suggesting stabilization of the AgNP-P colloidal solution [30].

The present study revealed the potent antibacterial effects against various *E. coli* strains (Uropathogens). To further understand the possible mechanism of antibacterial action, studies have shown that the combination between silver and propolis induces a 4–8 fold increase in antibacterial activity compared to individual components, primarily through cell wall disruption (reducing membrane integrity by 62%–75% as measured by propidium iodide uptake assays) and massive ROS generation (increasing intracellular ROS levels by 3.5-fold within 2 h) that damages bacterial DNA, proteins, and lipids [31,32]. The nanocomplex reduces biofilm formation by 78%–92% (crystal violet assays) and inhibits efflux pump activity (decreasing antibiotic efflux by 60% in ethidium bromide accumulation tests), effectively overcoming key resistance mechanisms in MDR strains [33]. Propolis enhances these effects through its high flavonoid content (12%–18% by HPLC analysis), which synergistically increases membrane permeability (electrolyte leakage assays show 85% more damage than silver nanoparticles alone) while suppressing virulent factors like pyocyanin production (reduced by 67%) [34]. This multi-targeted approach, combining physical membrane disruption (TEM shows complete cell wall lysis within 4 h) with biochemical oxidative damage (SOD and catalase activity reduced by 55%), presents a promising solution against resistant *P*. aeruginosa infections [35]. Toxins produced by some bacteria may have a role in the onset and progression of prostate cancer. For instance, the cell wall of bacteria like *E. coli* is mostly composed of lipopolysaccharide (LPS). The release of this substance occurs upon bacterial breakage; it is an endotoxin [35].

The synthesized NPP/AgONPs showed a significantly high selectivity index in HPrEC compared to PC-3 cells; the CC_50_ was 262.04 μg/mL, while the IC_50_ was 25.34 μg/mL, respectively. Researchers have discovered a strong correlation between LPS and the invasiveness of prostate cancer. The abnormal overexpression of a set of related genes, including those involved in cell proliferation, differentiation, and apoptosis, occurs when bacterial lipopolysaccharide (LPS) is continuously activated. This abnormal overexpression activates various pathways downstream of toll-like receptor 4 (TLR4), including the IL-6/STAT3, AKT/GSK-3β, and β-catenin pathways, which in turn induce the epithelial-mesenchymal transformation (EMT) of prostate cells [36]. The emission of reactive oxygen species and reactive nitrogen species occurs when phagocytes, primarily macrophages, are activated by components of bacteria. These elements have the potential to induce genetic instability by directly damaging DNA. Proliferative inflammatory atrophy (PIA) is a region that forms in the prostate as a result of induced oxidative stress and the cellular damage that follows. This area is a precursor to prostate cancer [37]. If pathogenic microbes are allowed to colonize undetected, they may alter or even eliminate the prostatic tissue’s native microbiome. This might cause an immune system imbalance and persistent inflammation [38]. Kumari et al. [39] formulated silver nanoparticles via bioactive fraction of *Pinus roxburghii* (PNb–AgONPs) which demonstrated pronounced cytotoxic effects on both A549 lung carcinoma cells and PC-3 prostate cancer cells, with IC_50_ values of 11.28 ± 1.28 μg/mL and 56.27 ± 1.17 μg/mL, respectively, while sparing normal human breast epithelial cells (fR2) and peripheral blood lymphocytes (PBL). In order to determine if there is a connection between cell cycle arrest and the inhibitory effects of the produced nanoparticles on cell growth, the distribution of PC-3 cells was studied using NPP/AgONPs. A similar study revealed that, when cultured in a prostate cancer cell line, *Chlamydia trachomatis* changed the levels of the proinflammatory cytokines IL-6 and FGF-2. They may be the primary mechanisms by which prostate cancer develops vascularization and metastatic lesions, as well as why it may resist treatment [40]. The chemokines IL-6, CCL2, and CXCL8, which are secreted in response to an infection with *Trichomonas vaginalis*, were shown to cause the M2 macrophages to become polarized, which in turn accelerated the growth of cancer [41]. According to Crocetto et al. [42], the infection with *T. vaginalis* may alter the microenvironment in a way that activates the epithelial-mesenchymal transition (EMT), which in turn increases the proliferation and invasiveness of prostate cancer cells [42]. Nanoparticles (NPs) produced by the *Salvia miltiorrhiza* plant may stop the growth of several bacteria, such as *S. aureus*, *Klebsiella pneumoniae*, *E. coli*, and *Bacillus subtilis*. To further explore silver nanoparticles’ antiprostate cancer potential, the LNCaP cell lines were used. The findings demonstrated that the synthesized nanoparticles caused cytotoxicity, reactive oxygen species (ROS), and cell death (apoptosis) in LNCap cell lines via regulating the production of intrinsic apoptotic proteins [24]. He et al. [43] described *in vitro* investigations on cytotoxicity effects utilizing the trypan blue assay on PC-3 cells, a model for human prostate cancer. Additional parameters measured were surviving, caspase 3, phosphorylated STAT3, and Bcl-2 levels. The procedure made extensive use of longan peel extract, a powerful reducing and stabilizing agent. The 9–32 nm water-soluble silver nanoparticles were gathered using a face-centered cubic configuration. Both gram-positive and gram-negative bacteria were killed by the nanoparticles, and the effectiveness of this bacterial cell inhibition was dosage dependent. Additionally, the synthesized nanoparticles demonstrated a dose-dependent cytotoxicity against PC-3 cells by increasing caspase 3 activity and decreasing the expression of STAT3, Bcl-2, and surviving [43]. The present study improved some key points of nanoparticles’ green synthesis, namely the lack of defined surface chemistry and reproducibility, lower antibacterial activity due to suboptimal metal loading/release, biocompatibility concerns and cytotoxicity toward normal cells, complexity, and lack of scalability and regulatory and reproducibility concerns due to complex plant mixtures (Table 3).

**Table 3:** Improvements in the current green synthesis of silver nanoparticles

Drawback in prior green synthesis reports	Present method of potential mitigation	Mechanistic/Design rationale
**Lack of defined surface chemistry and reproducibility**	Propolis coating provides more uniform surface chemistry and functional groups for stabilization	Propolis contains flavonoids, phenolics, and waxes that act as reducing and capping agents, promoting consistent stabilization
**Inconsistent particle size and aggregation**	Propolis-assisted decoration narrows size distribution and reduces aggregation	Propolis moieties offer steric and electrostatic stabilization, aiding dispersion
**Lower antibacterial activity due to suboptimal metal loading/release**	Synergistic enhancement from silver core + propolis matrix; potential tuned release	Propolis components may modulate Ag release and disrupt microbial membranes alongside NPP/AgONPs
**Biocompatibility concerns and cytotoxicity toward normal cells**	Propolis coating can tune biocompatibility and potentially improve selectivity	Propolis constituents modulate ROS and apoptosis pathways, potentially favoring cancer-cell targeting while reducing normal-cell damage in some contexts
**Complexity and lack of scalability**	Streamlined wet-chemical route using propolis extract; fewer steps	Propolis extract acts as both reducing and capping agent; reduces need for multiple reagents
**Stability in biological media and shelf life**	Propolis decoration enhances colloidal stability in physiological conditions	Phytochemicals provide steric and electrostatic stabilization; reduce protein corona-induced aggregation
**Regulatory and reproducibility concerns due to complex plant mixtures**	Defined propolis source with characterized composition improves traceability	Documenting extract composition and standardizing preparation reduces variability

However, the limitations of the present study may be the variability of propolis samples according to the geographical area and the climatic changes, which will affect the chemical composition, leading to variable activity.

## 5 Conclusions

The study demonstrates the use of a sustainable approach to synthesize NPP/AgONPs, which have a high crystallinity and stability. The nanoparticles have shown potent antibacterial effects against various *E. coli* strains, with *E. coli* 51 being the most resistant strain. Live/dead cell experiments were conducted to evaluate the proportion of growth decrease associated with an increase in red cells compared to green cells. The *in vitro* cytotoxic impact of the nanoparticles was examined using the HPrEC human normal prostate epithelial cell line. Moreover, the prepared nanoparticles effectively prevented and treated prostate cancer in PC-3, and flow cytometry analysis showed significantly higher rates of PC-3 cell apoptosis in the early, late, and death stages. The study also found that cell cycle arrest was associated with the growth-inhibitory effects of the synthetic nanoparticles.

## Data Availability

All the original data are available upon reasonable request for corresponding authors.
